# Optimizing oropharyngeal cancer management by using proton beam therapy: trends of cost-effectiveness

**DOI:** 10.1186/s12885-021-08638-2

**Published:** 2021-08-21

**Authors:** Guo Li, Yun-Fei Xia, Yi-Xiang Huang, Deniz Okat, Bo Qiu, Jerome Doyen, Pierre-Yves Bondiau, Karen Benezery, Jin Gao, Chao-Nan Qian

**Affiliations:** 1 Department of Radiation Oncology, Affiliated Cancer Hospital & Institute of Guangzhou Medical University, Guangzhou, Guangdong 510095 P. R. China; 2 State Key Laboratory of Oncology in South China and Collaborative Innovation Center for Cancer Medicine, Sun Yat-sen University Cancer Center, 651 Dongfeng East Road, Guangzhou, Guangdong 510060 P. R. China; 3 Department of Radiation Oncology, Sun Yat-sen University Cancer Center, Guangzhou, Guangdong 510060 P. R. China; 4 Department of Health Management, Public Health Institute of Sun Yat-sen University, Guangzhou, Guangdong 510000 P. R. China; 5 Department of Finance, Hong Kong University of Science and Technology, Kowloon, Hong Kong, P. R. China; 6 Department of Radiation Oncology, Antoine Lacassagne Cancer Center, University of Nice-Sophia, 06189 Nice, France; 7 Mediterranean Institute of Proton Therapy, Antoine Lacassagne Cancer Center, University of Nice-Sophia, 06200 Nice, France; 8 Department of Radiation Oncology, The First Affiliated Hospital of University of Science and Technology of China, Division of Life Sciences and Medicine, University of Science and Technology of China, Hefei, Anhui 230031 P. R. China; 9 Department of Radiation Oncology, Guangzhou Concord Cancer Center, Guangzhou, Guangdong 510045 P. R. China

**Keywords:** Oropharyngeal cancer, Proton beam therapy, Cost-effectiveness analysis, Intensity-modulated proton radiation therapy, Intensity-modulated photon-radiation therapy, Markov model, China

## Abstract

**Background:**

Proton beam therapy (PBT) is a new-emerging cancer treatment in China but its treatment costs are high and not yet covered by Chinese public medical insurance. The advanced form of PBT, intensity-modulated proton radiation therapy (IMPT), has been confirmed to reduce normal tissue complication probability (NTCP) as compared to conventional intensity-modulated photon-radiation therapy (IMRT) in patients with oropharyngeal cancer (OPC). Herein, we evaluated the cost-effectiveness and applicability of IMPT versus IMRT for OPC patients in China, aiming at guiding the proper use of PBT.

**Methods:**

A 7-state Markov model was designed for analysis. Base-case evaluation was performed on a 56-year-old (median age of OPC in China) patient under the assumption that IMPT could provide a 25% NTCP-reduction in long-term symptomatic dysphagia and xerostomia. Model robustness was examined using probabilistic sensitivity analysis, cohort analysis, and tornado diagram. One-way sensitivity analyses were conducted to identify the cost-effective scenarios. IMPT was considered as cost-effective if the incremental cost-effectiveness ratio (ICER) was below the societal willingness-to-pay (WTP) threshold.

**Results:**

Compared with IMRT, IMPT provided an extra 0.205 quality-adjusted life-year (QALY) at an additional cost of 34,926.6 US dollars ($), and had an ICER of $170,082.4/ QALY for the base case. At the current WTP of China ($33,558 / QALY) and a current IMPT treatment costs of $50,000, IMPT should provide a minimum NTCP-reduction of 47.5, 50.8, 55.6, 63.3 and 77.2% to be considered cost-effective for patient age levels of 10, 20, 30, 40 and 50-year-old, respectively. For patients at the median age level, reducing the current IMPT costs ($50,000) to a $30,000 level would make the minimum NTCP-reduction threshold for “cost-effective” decrease from 91.4 to 44.6%, at the current WTP of China (from 69.0 to 33.5%, at a WTP of $50,000 / QALY; and from 39.7 to 19.1%, at a WTP of $100,000 / QALY).

**Conclusions:**

Cost-effective scenarios of PBT exist in Chinese OPC patients at the current WTP of China. Considering a potential upcoming increase in PBT use in China, such cost-effective scenarios may further expand if a decrease of proton treatment costs occurs or an increase of WTP level.

**Supplementary Information:**

The online version contains supplementary material available at 10.1186/s12885-021-08638-2.

## Background

The worldwide incidence of oropharyngeal cancer (OPC) has increased in the past 40 years owing to the rising rates of human papillomavirus (HPV) infection related to sexual behavior [1]. Traditionally, China had a lower burden of HPV-positive OPC compared to the rest of the world. However, in the recent decade, changes in sexual behavior brought by high-pressure lifestyle have led to an upward trend in the occurrence of OPC in China [2]. As reported, during the years 2003–2012, the age-standardized rate of OPC incidence has increased from 2.0/100,000 per year to 2.54/100,000 per year [3].

According to the latest treatment guidelines of OPC, radiotherapy/concurrent chemoradiotherapy is recommended as the mainstream treatment for patients with early/locally advanced OPC [4, 5]. Owing to the need of a high irradiation dose for potential cure, localized adverse events such as irradiation-induced dysphagia and xerostomia become the most common late toxicities in OPC survivors. Even with the optimal photon irradiation technique, intensity-modulated photon-radiation therapy (IMRT), symptomatic dysphagia and xerostomia (grade 2–4) occur at incidences as high as 15–23% and 32–48%; and have been identified as independent negative factors impacting the long-term quality of life of OPC patients [6–9].

Proton beam therapy (PBT) is a new-emerging particle irradiation technique. Compared with conventional photon-radiation therapy, it has superior energy absorption distribution afforded by protons’ “Bragg peaks” to better protect normal tissues from radiation injury. The comparative dosimetric studies have confirmed that the intensity-modulated proton radiation therapy (IMPT, the advanced form of PBT) enabled to significantly reduce the irradiation dose to parotid, larynx, oral cavity, and pharyngeal constrictor muscle in OPC patients, thereby having the potential to reduce the normal tissue complication probability (NTCP) in long-term dysphagia and xerostomia [10–13]. In previous PBT decision-making studies, the ability of IMPT over IMRT in reducing dysphagia and xerostomia has been applied as a pivotal factor determining whether an OPC patient was appropriate to undergo PBT [14, 15].

Nevertheless, the therapeutic benefits of PBT should still be carefully weighed against its high treatment costs, which can be 3.2 to 4.8 times higher than that of IMRT [16]. On the mainland of China, there is currently only 1 operational proton center but the Chinese government has planned to authorize another 16 licenses for operating proton centers in the major cities by the year 2021 [17]. Meanwhile, the high treatment costs of this new-emerging cancer treatment are not yet covered by Chinese public medical insurance. As such, cost-effectiveness analysis (CEA) is urgently needed to ensure the proper use of PBT when this treatment would become more available in the near future [18, 19]. Till present, no CEA study has been conducted to evaluate the cost-effectiveness of PBT for Chinese OPC patients.

Herein, we designed a 7-state Markov model to track the natural development of OPC and late toxicities, and simultaneously evaluated the relevant cost and effectiveness of IMPT versus IMRT in a long-term period; aiming to identify cost-effective scenarios for optimal PBT efficacy in Chinese OPC patients.

## Methods

### Model design

The TreeAge Pro 2018 software (Williamstown, MA) was used for model building and analysis. A CEA model (a decision tree combining two-arm Markov model) was designed to evaluate the cost-effectiveness of IMPT, in comparison to that of IMRT, for newly diagnosed non-metastatic OPC patients. The CEA model was built based on the following two assumptions: (1) compared to IMRT, IMPT would be able to reduce symptomatic dysphagia and xerostomia (Radiation Therapy Oncology Group, RTOG grade 2–4), defined in this present study as “NTCP-reduction”; per the equation: NTCP-reduction (%) _=_ [(NTCP _after IMRT_ - NTCP _after IMPT_) / NTCP _after IMRT_] *100%; (2) all the dysphagia and xerostomia would occur within the first year after radiotherapy and the two symptoms would be irreversible once occurred [20, 21].

**Fig. 1:**
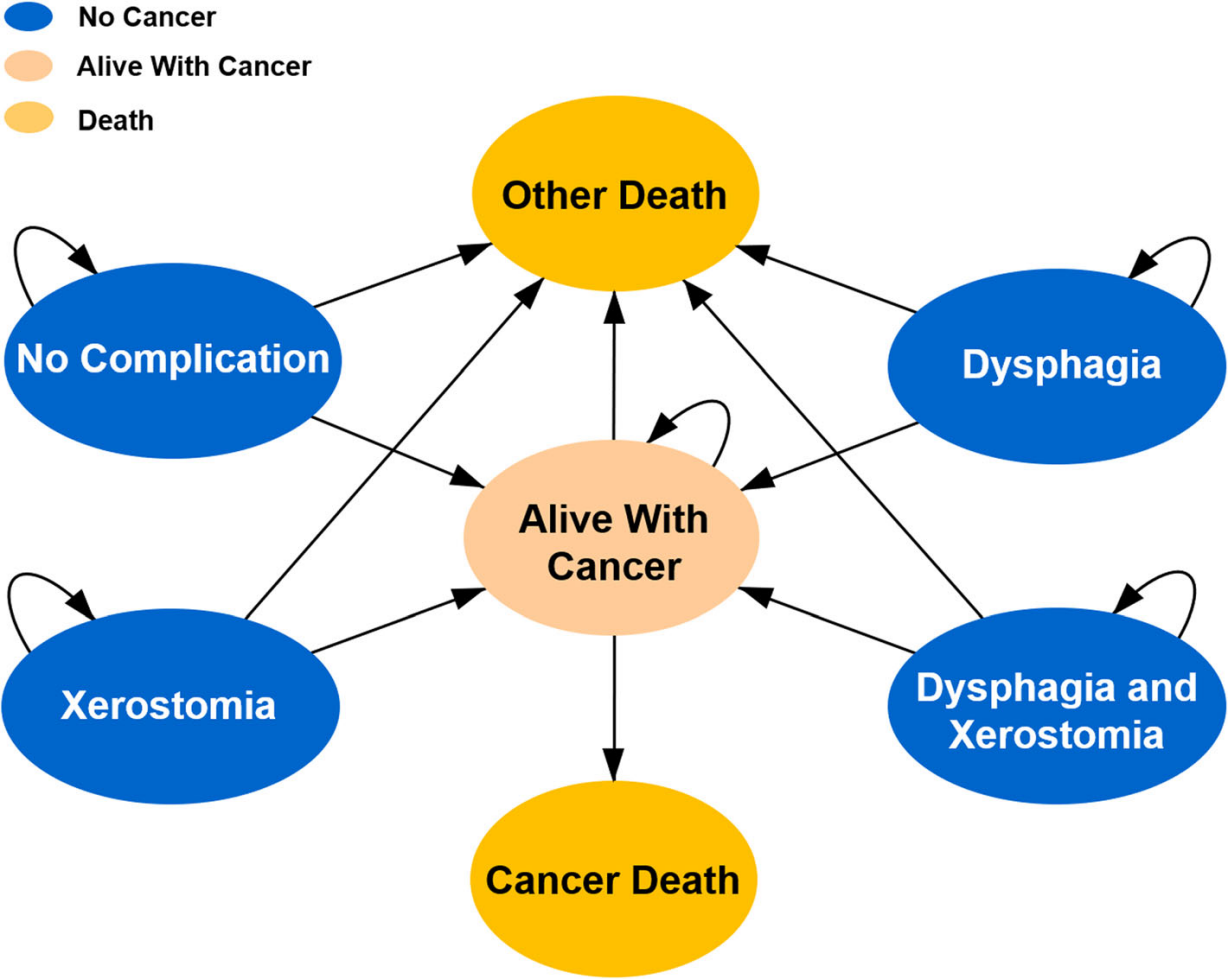
Transition states of the Markov model. Legend: The 3 main Markov states of “no cancer”, “alive with cancer” and “death” were used to simulate the disease process of oropharyngeal cancer. The state of “no cancer” included four sub-states, namely “dysphagia”, “xerostomia”, “dysphagia and xerostomia” and “no complication”. For each cycle, if the patient was in the state of “no cancer”, s/he might stay in the state of “no cancer”, develop into the state of “alive with cancer” or develop into the state of “death” (“other death”). If the patient was in the state of “alive with cancer”, s/he might stay in the state of “alive with cancer” or develop into the state of “death” (“cancer death” or “other death”). If the patient was in the absorbing state “death”, the loop operation would be terminated

Transition states of the Markov model are illustrated in Fig. 1. Different states, namely “alive with cancer”, “no cancer” (including 4 sub-states of “dysphagia”, “xerostomia”, “dysphagia and xerostomia” and “no complication”) and “death” (including “cancer death” or “other death”), were used to simulate the natural process of disease and late toxicities for OPC patients after radiotherapy. A 1-year cycle length was used, and the Markov models were cycled from 1 year after radiotherapy until the estimated generalized Chinese life expectancy (77 years old) for the year 2020, to evaluate the cost-effectiveness over a lifetime horizon [22]. Half-cycle corrections were performed to minimize discretization errors in the continuous Markov process. The risk of natural non-cancer deaths was calculated based on the United States 2016 Life Tables [23].

### Base-case set-ups

We assumed a 56-year-old (median age of OPC in China) male patient who had a squamous OPC of stage IVA (T2N2M0) as the base case to represent the Chinese OPC patients at the average level [24]. Disease probabilities in the model were calibrated according to the data of overall survival (OS) and disease-free survival (DFS), as reported by De Felice et al. [25]. The NTCPs of dysphagia and xerostomia after IMRT were set according to long-term follow-up results of OPC patients after IMRT, reported by Bird et al. and Al-Mamgani et al., in which the probabilities of dysphagia and xerostomia (≥ grade 2) were 0.19 and 0.33, respectively [5, 6]. On the basis of published toxicities data, we initially assumed that IMPT could provide a 25% NTCP-reduction in dysphagia and xerostomia compared with IMRT for the base case [12, 13]. Other clinical outcomes, including the survival rates, were assumed to be identical between the two strategies.

### Cost and utilities

The treatment regimens of the 2 compared strategies were similar except for the irradiation technique (IMPT versus IMRT), and included radical radiotherapy (32-fraction to a total dose of 70 Grays) and 3 cycles of concurrent chemotherapy. The cost of IMPT was estimated as being $50,000 based on the relevant charge standard applied in Shanghai Proton and Heavy Ion Center (Shanghai, China). Such treatment cost reflected both the capital investment (i.e. the estimates for building and infrastructure, hardware, dosimetry and engineering equipment, planning and clinical management software, and the working capital during the construction) and the operating costs (i.e. the estimates for land, equipment maintenance, electrical power, and the salaries for the staff). Another 2 assumed IMPT costs levels ($40,000 and $30,000) were applied to evaluate the influence of decrease in proton treatment costs on cost-effectiveness. The costs of IMRT were estimated as being $12,000 to reflect the similar treatment cost for photons irradiation at the Sun Yat-sen University Cancer Center (Guangzhou, China). The other costs for treatments and examinations were estimated based on casual clinical prescriptions to reflect similar costs as that of daily practice in a Chinese hospital. The cost of concurrent chemotherapy was assumed as $5000; simulating 3 cycles of 80–100 mg/m^2^ cisplatin bolus injection delivered on day-1, − 21, and − 42 of the radiotherapy. The follow-up cost per year was assumed as $1000; simulating a set of examinations including hematologic and biochemistry profiles, nasopharyngeal fiberoptic endoscope examination, magnetic resonance imaging of head and neck, chest radiography, and abdominal ultrasonography. The cost of palliative therapy per year was assumed as $5000; simulating 8 cycles of oral palliative chemotherapy based on 5-fluorouracil. The treatment cost for dysphagia per year was estimated as $3000; simulating the long-term requirement of a nasal feeding tube or percutaneous gastrostomy tube. The treatment cost for xerostomia per year was estimated as $2000; simulating the use of traditional Chinese medicine/artificial saliva and additional intensive dental care. For patients in the “no cancer” state, the annual cost included the follow-up cost ± the treatment cost for dysphagia and/or xerostomia. For patients in the “alive with cancer” state (recurrence, metastasis, or residue), the annual cost included the follow-up cost and the cost of palliative chemotherapy. All costs were adjusted to US dollars ($), using a Sino-US exchange rate of $1 = 6.47 RMB (February 28, 2021).

The utilities were adjusted to QALY using health state utility values (HSUV). On the basis of published data, the HSUV of “alive with cancer” was assumed to be 0.57, representing a progressive disease with the disutility caused by anticancer treatment [26]. The HSUVs for the states of “dysphagia”, “xerostomia” and “dysphagia and xerostomia” (≥ grade 2) were standardized as being 0.803, 0.846, and 0.763 [9]. The HSUVs for the states of “no complication” and “death” were set as 1 and 0. Costs and quality-adjusted life-year (QALY) were discounted at an annual rate of 3% [27].

### Sensitivity analysis and Monte Carlo simulation

Probabilistic sensitivity analysis was applied to illustrate the robustness of the model in light of a joint uncertainty for model parameters by running over 50,000 iteration trials, and the 90% confidence interval of the model parameters were identified. Tornado diagram was used to evaluate the influences of the parameters on the ICER over the variation of their 90% confidence interval. One-way sensitivity analyses were performed to identify cost-effective threshold values for the parameters. Monte Carlo simulation (50,000 trials) was applied to show the trials distributions of the two strategies and to determine the recommended strategy from the perspective of net benefit.

### Outcome measurement

OS was defined as the time interval between the end of the radiotherapy and death from any cause. DFS was defined as the time interval between the end of the radiotherapy and first cancer progression or death from any cause. The outcome measure of the model was the incremental cost-effectiveness ratio (ICER) which represented the ratio of the difference in costs to the difference in effectiveness (incremental cost / incremental effectiveness) between IMPT and IMRT. A strategy was deemed cost-effective by comparing the ICER of the strategy with an established societal willingness-to-pay (WTP). According to the World Health Organization guidelines, a strategy is defined as cost-effective if the ICER value is below 3 times the gross domestic product (GDP) per capita [28]. In this study, $33,558 / QALY (3 times the Chinese GDP per capita in 2020) was applied as the current WTP level of China; and 2 common WTP thresholds ($50,000/QALY and $100,000/QALY) were applied to evaluate future trends [29].

## Results

### Model robustness verification

**Fig. 2:**
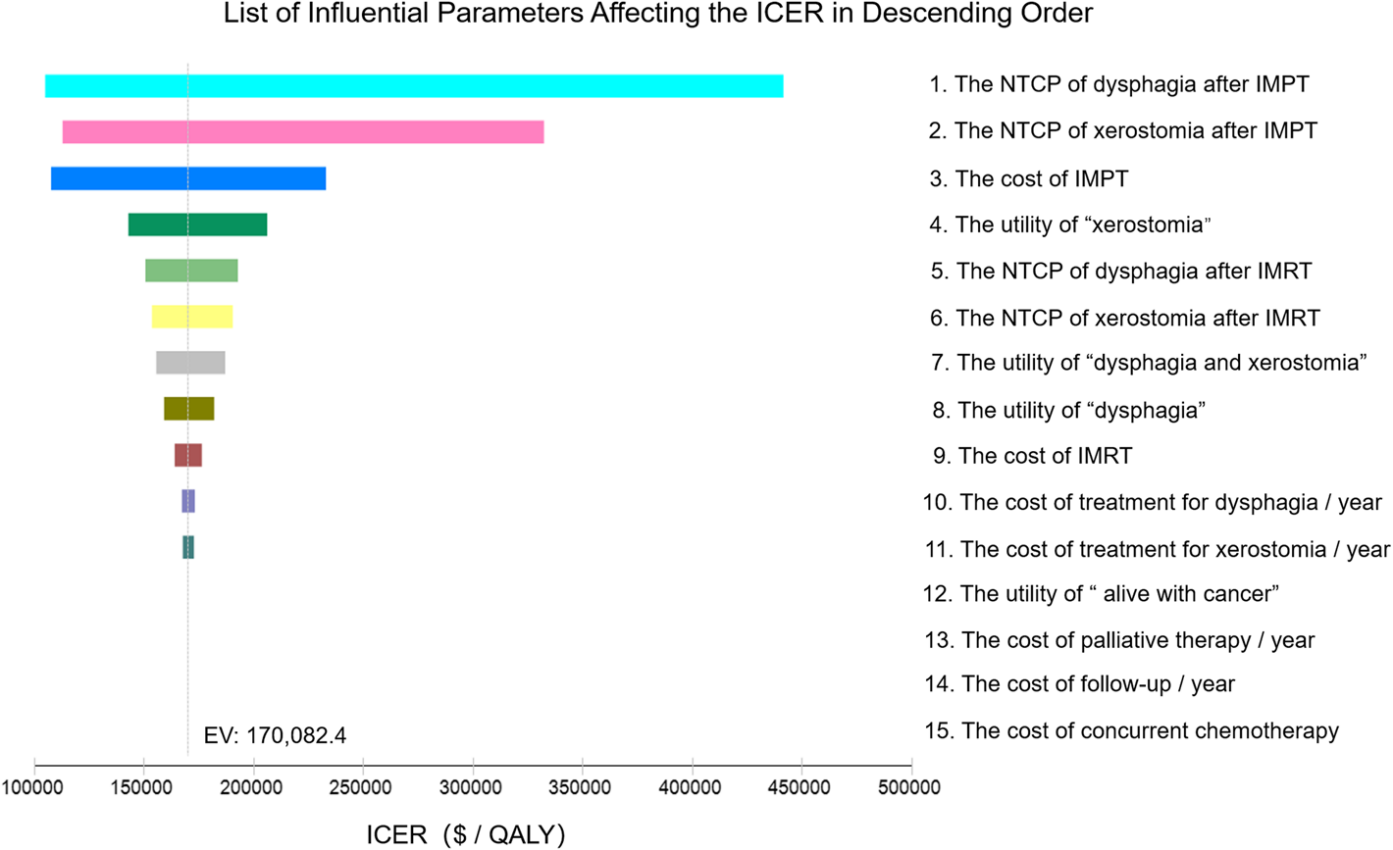
Tornado diagram illustrating the influential parameters affecting the incremental cost-effectiveness ratio. Legend: The tornado diagram demonstrates the range of incremental cost-effectiveness ratio (ICER) when varying each parameter individually. Influential parameters are listed in descending order according to their abilities to affect the ICER over the variation of their 90% confidence interval. IMPT, intensity-modulated proton radiation therapy; IMRT, intensity-modulated photon-radiation therapy; NTCP, normal tissue complication probability; EV, expected value; QALY, quality-adjusted life-year; $, US dollars

The input information and probabilistic sensitivity analyses for the model parameters are summarized in Table 1. The model robustness verification was performed using the base-case set-ups. Markov cohort analyses for both IMPT and IMRT strategy are shown in Additional file 1: Fig. S1. The CEA model for the base case predicted a 1-,3-, 5- and 10-year OS rates of 90.0, 85.5, 80.0 and 68.7%, and the 1-,3-, 5- and 10-year DFS rates of 85.0, 78.8, 72.9 and 67.1%, respectively (detailed in Additional file 2: Fig. S2). The tornado diagram identified the NTCP of dysphagia after IMPT, the NTCP of xerostomia after IMPT and the cost of IMPT as the top 3 parameters influencing the ICER. The other parameters had only a minor impact on the ICER (Fig. 2).

**Table 1:** Model information and probabilistic sensitivity analysis for the analyzed parameters

Parameters	Input Information	90% CI in PSA^a^	Distribution^b^	Source
**Target cancer**	OPC			
**Evaluated treatment strategies**	IMPT vs. IMRT			
**Base-case set-ups**				
Patient age^c^	56-year-old^d^			
Disease probabilities				
DFS (1-year)	0.85			De Felice et al. [25]
OS (1-year)	0.9			De Felice et al. [25]
“no cancer” to “alive with cancer”	0.03(2nd-5rd year); 0 (6th -10th year)			De Felice et al. [25]
“alive with cancer” to “cancer death”	0.3			De Felice et al. [25]
Long-term toxicities probabilities				
NTCP of dysphagia after IMRT	0.19	0.129–0.256	Beta	Bird et al. [5]
NTCP of xerostomia after IMRT	0.33	0.266–0.394	Beta	Al-Mamgani et al. [6]
NTCP of dysphagia after IMPT	0.143	0.082–0.209	Beta	Meijer et al. [12]
NTCP of xerostomia after IMPT	0.248	0.185–0.312	Beta	Meijer et al. [12]
**Utilities (QALY)**				
No complication	1			
Dysphagia	0.803	0.671–0.918	Beta	Ramaekers et al. [9]
Xerostomia	0.846	0.714–0.955	Beta	Ramaekers et al. [9]
Dysphagia and xerostomia	0.763	0.631–0.881	Beta	Ramaekers et al. [9]
Alive with cancer	0.57	0.442–0.696	Beta	de Almeida et al. [26]
Death (cancer death or other death)	0			
**Cost ($)**				
IMPT	50,000	37,134.7 - 62,882.3	Normal	SPHIC
IMRT	12,000	10,737.4 - 13,282.5	Normal	SYSUCC
Concurrent chemotherapy	5000	3728.8 - 6297.3	Normal	SYSUCC
Follow-up / year	1000	869.7–1128.0	Normal	SYSUCC
Treatment for dysphagia / year	3000	1716.6 - 4273.0	Normal	SYSUCC
Treatment for xerostomia / year	2000	1362.6 - 2641.6	Normal	SYSUCC
Palliative therapy / year	5000	2411.2 - 7567.2	Normal	SYSUCC
**Markov model set-up**				
Cycle length	1-year			
Number of cycles	77^e^ - patient age			
Discount rate / year	3%			

*CI* confidence interval, *PSA* probabilistic sensitivity analysis, *OPC* oropharyngeal cancer, *IMPT* intensity-modulated proton radiation therapy, *IMRT* intensity-modulated radiation therapy, *OS* overall survival, *DFS* disease-free survival, *NTCP* normal tissue complication probability, *QALY* quality-adjusted life-year, *$* US dollars, *SPHIC* Shanghai Proton and Heavy Ion Center, *SYSUCC* Sun Yat-sen University Cancer Center, *CEA* cost-effectiveness analysis

^a^Probabilistic sensitivity analysis (PSA) was performed to determine 90% CI for model parameters by running over 50,000 iteration trials

^b^The utilities and probabilities were tested using beta distribution and the costs were tested using uniform distribution

^c^The patient age when preparing for radiotherapy

^d^Median age of OPC patients in China

^e^The estimated Chinese life expectancy. For the base case, Markov models were to be cycled 21 times to evaluate the outcomes over a time-period from 1 year after radiotherapy to the end of the estimated Chinese life expectancy

### Cost-effectiveness of the base case

By model calculation, IMPT (compared with IMRT) provided the base case an additional 0.205 QALY at an additional cost of $34,926.6, and the ICER was $170,082.4/QALY. Thus, IMPT was not cost-effective for the base case at the current WTP of China ($33,558/QALY). In the Monte Carlo simulations, IMPT was the recommended strategy in 32.7% of trials from the perspective of net benefit (Additional file 3: Fig. S3). One-way sensitivity analyses with base-case set-ups identified cost-effective threshold values for the parameters using 3 different WTP thresholds ($33,558 / QALY, $50,000 / QALY, and $100,000 / QALY) (Additional file 4: Table S1). IMPT could be cost-effective for the base case if IMPT treatment costs ($50,000) reduced to ≤ $ 21,964.6, ≤ 25,341.0 and ≤ 35,608.5 at the WTP thresholds of $33,558 / QALY, $50,000 / QALY and $100,000 / QALY, respectively.

### Stratified analyses

Stratified analyses were conducted for NTCP-reduction levels and age levels. The ICERs under different NTCP-reduction levels and different age levels are listed in Table 2. For OPC patients at median age level (56-year-old), the corresponding ICERs were $454,083.8 / QALY, $217,370.3 / QALY, $138,587.0 / QALY, $99,283.6 / QALY, $75,770.2 / QALY, $65,150.4 / QALY, $49,049.7 / QALY and $40,746.3 / QALY under the NTCP-reduction levels of 10, 20, 30, 40, 50, 60,70 and 80%, respectively.

**Table 2:** Incremental cost-effectiveness ratio under different NTCP-reduction levels and age levels

NTCP-reduction^a^	Incremental Cost-effectiveness Ratio ($ / QALY) ^b^													
	10-year-old		20-year-old		30-year-old		40-year-old		50-year-old		60-year-old		70-year-old	
	IC / IE	ICER	IC / IE	ICER	IC / IE	ICER	IC / IE	ICER	IC / IE	ICER	IC / IE	ICER	IC / IE	ICER
10%	35,500.8 / 0.16	224,596.4	35,671.1 / 0.15	241,462.0	35,883.7 / 0.13	266,157.7	36,161.8 / 0.12	306,609.3	36,518.4 / 0.10	379,253.1	36,957.9 / 0.07	530,943.8	37,524.3 / 0.04	1,065,357.4
20%	33,001.6 / 0.32	103,402.8	33,342.1 / 0.30	111,778.5	33,767.4 / 0.27	124,042.8	34,323.5 / 0.24	144,131.8	35,036.8 / 0.19	180,208.0	35,915.8 / 0.14	255,540.4	37,048.7 / 0.07	520,939.7
30%	30,502.4 / 0.48	63,116.3	31,013.2 / 0.45	68,662.8	31,651.1 / 0.41	76,784.4	32,485.3 / 0.36	90,087.6	33,555.2 / 0.29	113,977.6	34,873.8 / 0.21	163,863.6	36,573.0 / 0.11	339,614.2
40%	28,003.2 / 0.65	43,054.3	28,684.2 / 0.61	47,186.7	29,534.7 / 0.55	53,237.7	30,647.0 / 0.49	63,149.2	32,073.6 / 0.40	80,948.4	33,831.7 / 0.29	118,115.9	36,097.4 / 0.14	249,058.6
50%	25,504.0 / 0.82	31,080.2	26,355.3 / 0.77	34,364.6	27,418.4 / 0.70	39,173.8	28,808.8 / 0.61	47,051.3	30,592.0 / 0.50	61,197.8	32,789.6 / 0.36	90,737.8	35,621.7 / 0.18	194,808.7
60%	23,004.8 / 0.99	23,148,.8	24,026.3 / 0.93	25,868.1	25,302.1 / 0.85	29,849.9	26,970.5 / 0.74	36,372.2	29,110.4 / 0.61	48,085.0	31,747.5 / 0.44	72,542.9	35,146.1 / 0.22	158,709.6
70%	20,505.6 / 1.17	17,526.1	21,697.4 / 1.09	19,842.1	23,185.8 / 1.00	23,233.4	25,132.3 / 0.87	28,788.3	27,628.7 / 0.71	38,763.8	30,705.5 / 0.52	59,594.3	34,670.4 / 0.26	132,980.9
80%	18,006.4 / 1.35	13,345.5	19,368.4 / 1.26	15,359.2	21,069.5 / 1.15	18,307.9	23,294.0 / 1.01	23,137.8	26,147.1 / 0.82	31,811.5	29,663.4 / 0.59	49,923.4	34,194.8 / 0.30	113,732.3

*NTCP* normal tissue complication probability, *IC* incremental cost, *IE* incremental effectiveness, *ICER* incremental cost-effectiveness ratio, *$* US dollars, *QALY* quality-adjusted life-year, *IMPT* intensity-modulated proton radiation therapy, *IMRT* intensity-modulated photon-radiation therapy

^a^NTCP-reduction referred to the advantage of IMPT over IMRT in reducing symptomatic dysphagia and xerostomia, and calculated with the equation: NTCP-reduction (%) _=_ [(NTCP _after IMRT_ - NTCP _after IMPT_) / NTCP _after IMRT_] *100%

^b^The outcome measure of cost-effectiveness is ICER (ICER = IC [$] / IE [QALY])

### Cost-effective scenarios and trends

**Fig. 3:**
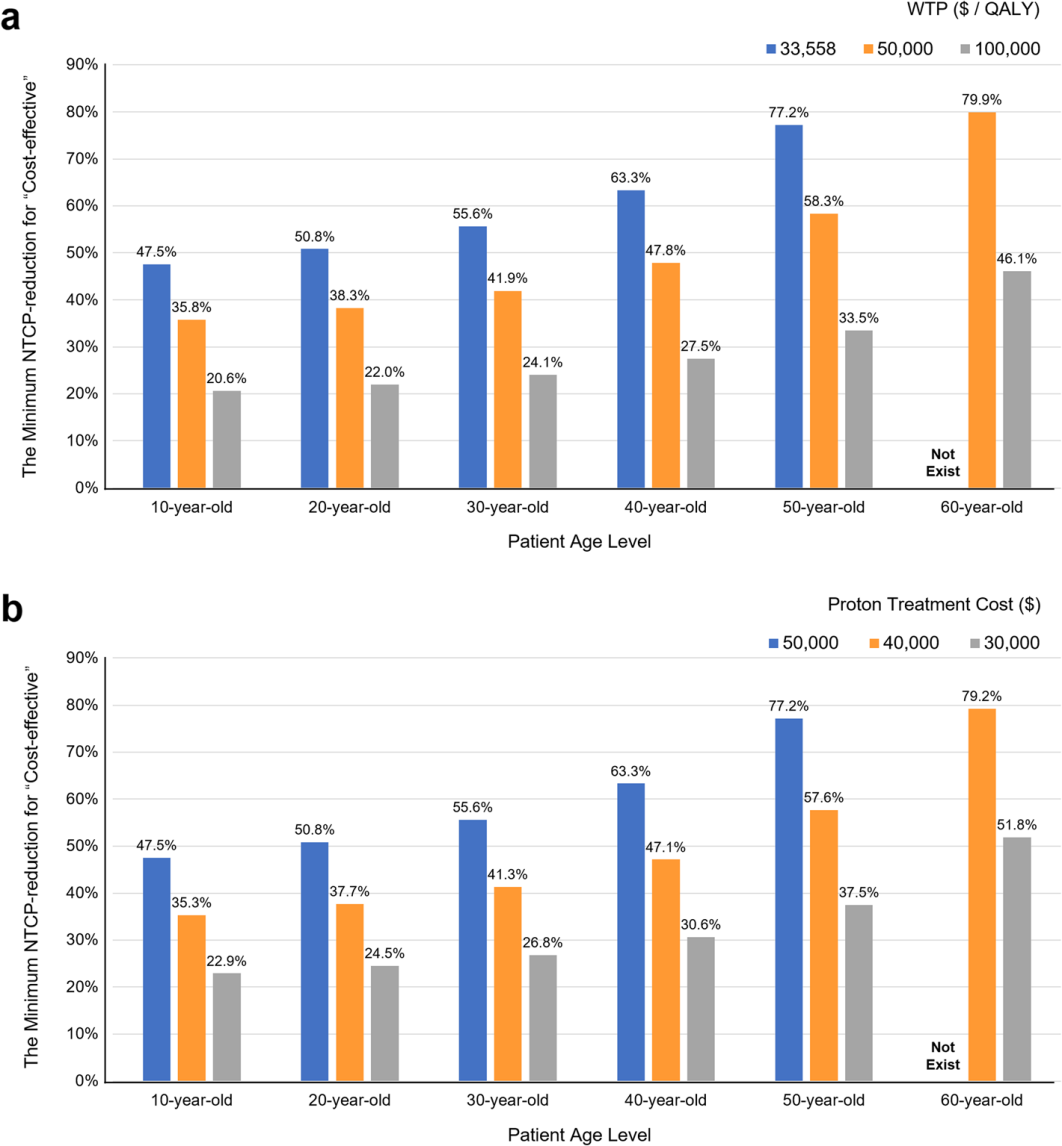
Cost-effective scenarios and trends. **a**. The minimum NTCP-reduction for “cost-effective” at different WTP levels. **b**. The minimum NTCP-reduction for “cost-effective” at different proton treatment cost levels. Legend: IMPT becomes cost-effective when the NTCP-reduction is ≥ the corresponding percentage shown on the top of the bar. IMPT, intensity-modulated proton radiation therapy; NTCP, normal tissue complication probability; WTP: willingness-to-pay; $, US dollars; QALY, quality-adjusted life-year

With different set-ups for WTP levels ($33,558 / QALY, $50,000 / QALY and $100,000 / QALY) and proton treatment cost levels ($50,000, $40,000 and $30,000), one-way sensitivity analyses identified the minimum NTCP-reduction threshold, above which IMPT could be cost-effective, for different patients age levels, as shown in Additional file 5: Table S2. At the current WTP of China ($33,558 / QALY) and a IMPT treatment costs level of $50,000, IMPT should provide a minimum NTCP-reduction of 47.5, 50.8, 55.6, 63.3 and 77.2% to be considered cost-effective for patient age levels of 10, 20, 30, 40 and 50-year-old, respectively. The cost-effective thresholds of NTCP-reduction decreased significantly with the growth of WTP level and the reduction of proton treatment costs (Fig. 3). For OPC patients at median age level (56-year-old), reducing the current IMPT costs ($50,000) to a $30,000 level would make the minimum NTCP-reduction threshold for “cost-effective” decrease from 91.4 to 44.6%, at the current WTP of China (decrease from 69.0 to 33.5%, at a WTP of $50,000 / QALY; and decrease from 39.7 to 19.1%, at a WTP of $100,000 / QALY).

## Discussion

In this study, a reliable and robust 7-state Markov model was designed to evaluate the cost-effectiveness of IMPT versus IMRT for OPC, whose incidence is fast-growing in China. Base-case evaluation was performed to examine the model robustness and evaluate cost-effectiveness at median age level, the minimum NTCP-reduction threshold for “cost-effective” were identified to evaluate the cost-effective scenarios. Our analyses demonstrated that the cost-effective scenarios of PBT currently exist in Chinese OPC younger patients who could obtain profound NTCP-reduction from PBT; such cost-effective scenarios largely expanded with the improvement of the WTP level and the decrease of proton treatment costs.

The cost-effectiveness of PBT has been poorly evaluated worldwide and has been referred to as the proton’s “economic controversy” [18, 30]. The only documented CEA study on PBT for OPC patients was reported by Sher et al. [31], who designed a 6-state Markov model using the base case of a 65-year-old OPC patient with American hospital settings, on the assumption that IMPT could provide a 25% reduction in xerostomia, dysgeusia and the need for a gastrostomy tube. However, the established 6-state Markov model by Sher et al. [31] was found inapplicable for Chinese OPC patients in regards to the preferential use of nasal feeding tube, rather than percutaneous gastrostomy tube, for treating eating difficulty, and the use of traditional Chinese medicine when dealing with xerostomia by Chinese patients.

The Markov model design is a key step of CEA modeling for PBT. In our previous CEA modeling for paranasal sinus and nasal cavity cancer, a reliable 3-state Markov model was designed to simulate the tumor development and evaluate the cost-effectiveness of IMPT in comparison to IMRT in terms of tumor control improvement [32]. Unlike paranasal sinus and nasal cavity cancer, the advantages of IMPT over IMRT for OPC patients was the reduction of late toxicities whilst no improvement in tumor control or survival rates [33, 34]. In this CEA modeling for OPC, we similarly used 3 main states including “alive with cancer”, “no cancer” and “death” to simulate the tumor development of OPC, but the survival probabilities were set identical between IMPT and IMRT strategy. The model-predicted OS and DFS were found to correspond to the previous long-term survival outcomes reported by De Felice et al. [25], which demonstrated that the CEA modeling indeed followed the natural disease process of OPC.

To evaluate the long-term differences in effectiveness and cost between the two treatment strategies, the state of “no cancer”, which refers to OPC survivor after radiotherapy, was further divided into 4 sub-states (“dysphagia”, “xerostomia”, “dysphagia and xerostomia” and “no complication”). The initial state probabilities of the 4 sub-states were set according to the NTCPs of dysphagia and xerostomia. The observed HSUVs for the “dysphagia”, “xerostomia”, “dysphagia and xerostomia” and “no complication” sub-states were set as 0.803, 0.846, 0.763, and 1, respectively, and the annual treatment costs for these late toxicities were added as the accumulated incremental costs. Therefore, NTCP-reduction (superiority of IMPT over IMRT in reducing dysphagia and xerostomia) became the motivating force to examine the differences of cost and effectiveness between the two strategies. The robustness of this model design was confirmed using tornado diagram analysis, which demonstrated that only the NTCPs after IMPT and the cost of IMPT had major impacts on ICER value.

NTCP-reductions and patient’s age level were two pivotal factors determining whether IMPT was cost-effective to an OPC patient in our CEA modeling. Thus, we conducted stratified analyses for NTCP-reduction levels and age levels, based on which the cost-effective threshold for NTCP-reduction was more readily identified for different patients age levels. With the current WTP of China ($33,558 / QALY) and a proton treatment costs of $50,000, PBT was found more favorable to the younger OPC patients who could obtain a profound NTCP-reduction. These findings could provide clinical insight in terms of guiding the most cost-effective use of this limited and expensive irradiation technique in Chinese OPC patients setting. But it should be noted that the cost-effectiveness of PBT would likely change with the potential upcoming growth of PBT use in China. In our analyses using different set-ups for WTP levels and proton treatment cost levels, the cost-effective thresholds of NTCP-reduction were found decreased significantly with the growth of WTP level and the reduction of proton treatment costs. With more proton centers opening in China in the near future, technology upgrades and market competition would likely promote size reduction in proton facility layout and increase in patient throughput efficiency, thereby contributing to a gradual decrease of proton treatment costs. A 20% or 40% reduction to the current high costs ($50,000), the hypothetical scenarios in our analyses, may occur [35, 36]. Meanwhile, the WTP level might increase with gradual increment of medical insurance coverage (public or private) and economic growth. As such, we estimated that the cost-effective scenarios of PBT for OPC patients would expand along with these future changes.

There were two limitations to this study worth mentioning. First, our CEA modeling was assumption-based. We assumed that all the symptomatic dysphagia and xerostomia would occur within the first year after radiotherapy and late toxicities would be irreversible once occurred. This assumption was made based on the previously observed studies, which showed that the risk of these two late toxicities would be high in the first year but negligible thereafter [37, 38]. Besides, the potential occurrence of dysphagia or xerostomia were not evaluated for a small part of patients in the state of “alive with cancer”, for whom we assumed that the main suffering should be their progressive cancer and anticancer treatment. Hence, these assumption-related problems may hamper the interpretation of the results to a certain extent. Second, the CEA modeling in this study was performed on a series of assumed set-ups. However, the clinical decision making of using PBT to a specific OPC patient should be based on CEA modeling with an individualized set-up, which enables to take the patient’s age and the specific therapeutic benefits from IMPT into account. Thus, we plan to create an individualized CEA modeling to guide treatment decisions for real OPC patients.

## Conclusions

On the basis of published data and assumption-based CEA modeling, the analyses of this study demonstrated that cost-effective scenarios of PBT do exist in Chinese OPC patients at the current WTP of China. At current stage, PBT is more favorable to the younger OPC patients who could obtain a profound NTCP-reduction. With emerging novel proton technologies optimizing therapeutic benefits, gradual increment of medical insurance coverage, economic growth, and proton treatment costs reduction due to the opening of more proton centers in China, PBT could become more cost-effective.

## Supplementary Information

**Additional file 1: Figure S1.** Markov cohort analyses. **a.** Cohort analysis for intensity-modulated proton radiation therapy strategy. **b.** Cohort analysis for intensity-modulated photon-radiation therapy strategy. **Legend:** Markov state probabilities of the base case were calculated in the cohort analyses for both intensity-modulated proton radiation therapy (IMPT) strategy and intensity-modulated photon-radiation therapy (IMRT) strategy.

**Additional file 2: Figure S2.** Model-predicted survival data. **Legend:** The model-predicted survival rates for the base case were in comparison with the previous outcomes reported by De Felice et al. [25]. The CEA model predicted the 2-,3- and 5-year OS rates of 87.8, 85.5 and 80.0%, in comparison with the previous 2-,3- and 5-year OS rates of 86.8, 84.6 and 78.1%; and the 3- and 5-year DFS rates of 78.8 and 72.9%, in comparison with the previous 3- and 5-year DFS rates of 78.6 and 73.9%. OS, overall survival; DFS, disease-free survival.

**Additional file 3: Figure S3.** Monte Carlo simulations. **a.** Incremental cost-effectiveness scatter plot (trials distribution). **b.** Strategy selection chart. **Legend:** Monte Carlo simulation (with 50,000 trials) was performed with the base-case set-ups at the WTP of $33,558 / QALY. **a,** each point represented 1 of those simulations and was charted at the simulation’s resultant incremental cost versus incremental effectiveness of IMPT compared with IMRT. **b,** strategy selection from the perspective of net benefit demonstrated that only 32.7% of trials favored IMPT to IMRT. $, US dollars; IMPT, intensity-modulated proton radiation therapy; IMRT, intensity-modulated photon-radiation therapy; QALY, quality-adjusted life-year; WTP: willingness-to-pay.

**Additional file 4: Table S1** One-way sensitivity analyses with the base-case set-up.

**Additional file 5: Table S2** One-way sensitivity analysis identifying the cost-effective threshold value for NTCP-reduction.

## Data Availability

The datasets used and analyzed during the current study are available from the corresponding author on reasonable request.

## Abbreviations

PBT: Proton beam therapy
IMPT: Intensity-modulated proton radiation therapy
IMRT: Intensity-modulated photon-radiation therapy
OPC: Oropharyngeal cancer
NTCP: Normal tissue complication probability
PSA: Probabilistic sensitivity analysis
ICER: Incremental cost-effectiveness ratio
WTP: Willingness-to-pay
HPV: Human papillomavirus
CEA: Cost-effectiveness analysis
RTOG: Radiation Therapy Oncology Group
OS: Overall survival
DFS: Disease-free survival
GDP: Gross domestic product
$: US dollars
QALY: Quality-adjusted life-year
SPHIC: Shanghai proton and heavy Ion Center
SYSUCC: Sun Yat-sen University Cancer Center
HSUV: Health state utility value
