# Supplementary figures and images for: Optimizing oropharyngeal cancer management by using proton beam therapy: trends of cost-effectiveness
Source: BMC Cancer. 2021 Aug 21;21:944. doi: 10.1186/s12885-021-08638-2 (PMC8380358; doi:10.1186/s12885-021-08638-2)

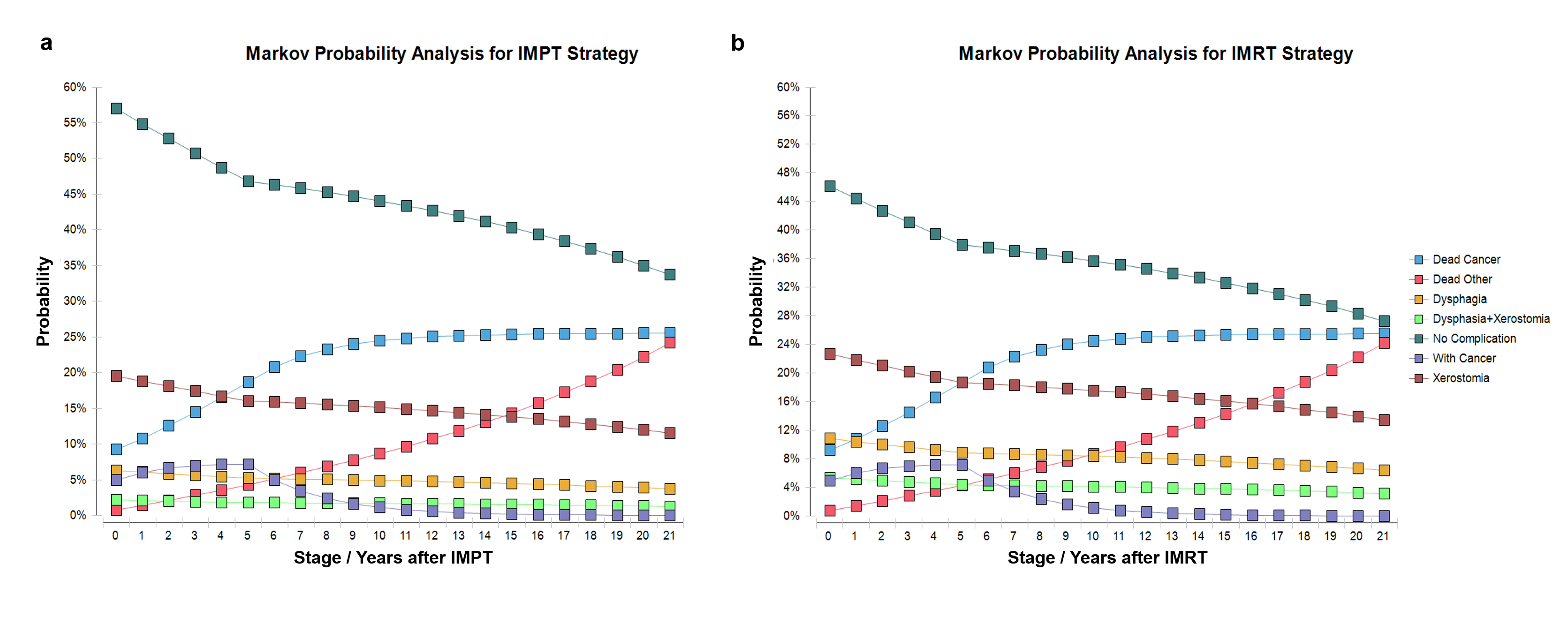

Supplement: Supplementary file 1 — Additional file 1: Figure S1. Markov cohort analyses. a. Cohort analysis for intensity-modulated proton radiation therapy strategy. b. Cohort analysis for intensity-modulated photon-radiation therapy strategy. Legend: Markov state probabilities of the base case were calculated in the cohort analyses for both intensity-modulated proton radiation therapy (IMPT) strategy and intensity-modulated photon-radiation therapy (IMRT) strategy. [file 12885_2021_8638_MOESM1_ESM.tif]

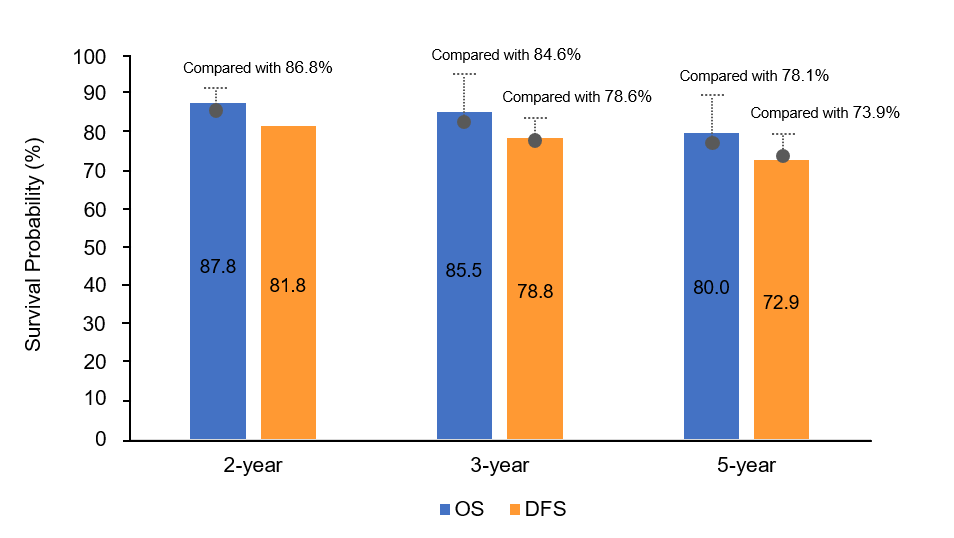

Supplement: Supplementary file 2 — Additional file 2: Figure S2. Model-predicted survival data. Legend: The model-predicted survival rates for the base case were in comparison with the previous outcomes reported by De Felice et al. [25]. The CEA model predicted the 2-,3- and 5-year OS rates of 87.8, 85.5 and 80.0%, in comparison with the previous 2-,3- and 5-year OS rates of 86.8, 84.6 and 78.1%; and the 3- and 5-year DFS rates of 78.8 and 72.9%, in comparison with the previous 3- and 5-year DFS rates of 78.6 and 73.9%. OS, overall survival; DFS, disease-free survival. [file 12885_2021_8638_MOESM2_ESM.tif]

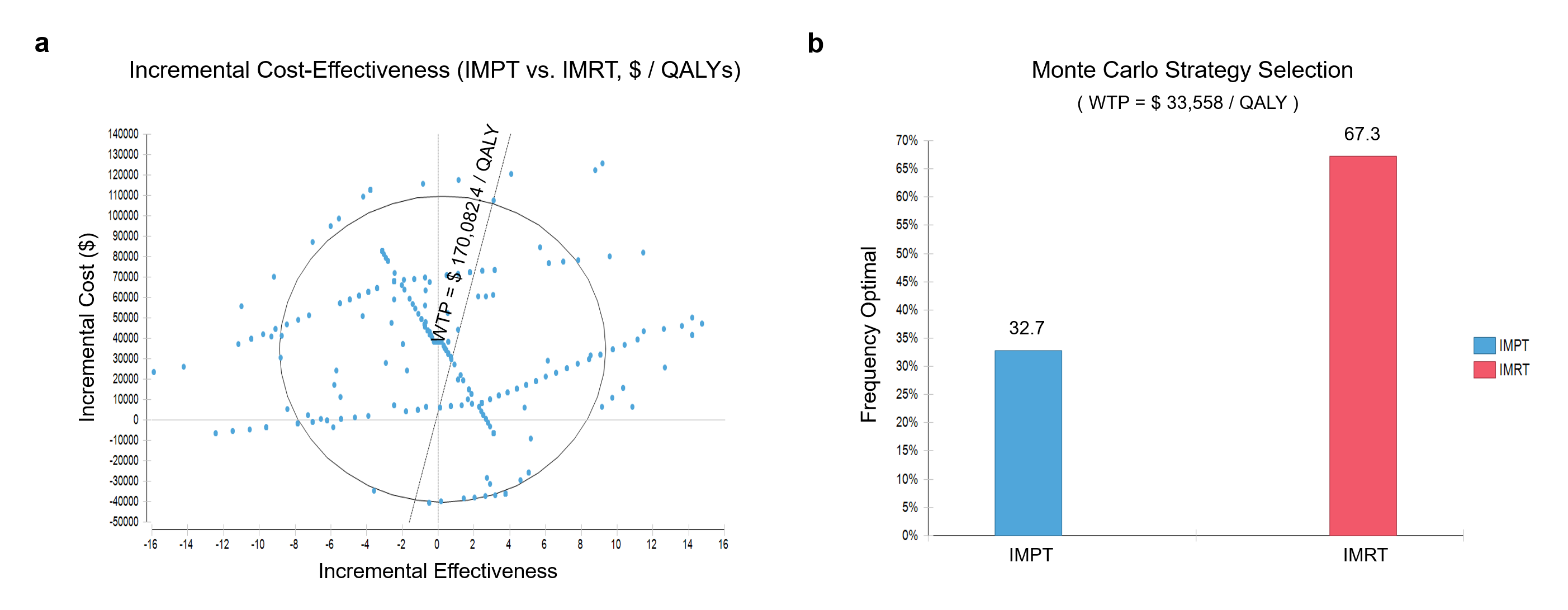

Supplement: Supplementary file 3 — Additional file 3: Figure S3. Monte Carlo simulations. a. Incremental cost-effectiveness scatter plot (trials distribution). b. Strategy selection chart. Legend: Monte Carlo simulation (with 50,000 trials) was performed with the base-case set-ups at the WTP of $33,558 / QALY. a, each point represented 1 of those simulations and was charted at the simulation’s resultant incremental cost versus incremental effectiveness of IMPT compared with IMRT. b, strategy selection from the perspective of net benefit demonstrated that only 32.7% of trials favored IMPT to IMRT. $, US dollars; IMPT, intensity-modulated proton radiation therapy; IMRT, intensity-modulated photon-radiation therapy; QALY, quality-adjusted life-year; WTP: willingness-to-pay. [file 12885_2021_8638_MOESM3_ESM.tif]
