# Supplementary material for: Optimizing oropharyngeal cancer management by using proton beam therapy: trends of cost-effectiveness
Source: BMC Cancer. 2021 Aug 21;21:944. doi: 10.1186/s12885-021-08638-2 (PMC8380358; doi:10.1186/s12885-021-08638-2)
Supplement: Supplementary file 4 — Additional file 4: Table S1 One-way sensitivity analyses with the base-case set-up. [file 12885_2021_8638_MOESM4_ESM.docx]

**Additional File 4 Table S1** One-way sensitivity analyses with the base-case set-up

| Parameters | Base Case Set-up | Expected  Value | Cost-effective Threshold Value | | | |
| --- | --- | --- | --- | --- | --- | --- |
|  |  |  | $33,558 / QALY^b^ | | $50,000 / QALY | $100,000 / QALY |
| NTCP-reduction^a^ | 25% | Minimum | 91.4% | 69.0% | | 39.7% |
| The cost of IMPT ($) | 50,000 | Maximum | 21,964.6 | 25,341.0 | | 35,608.5 |

*NTCP* normal tissue complication probability, *IMPT* intensity-modulated proton radiation therapy, *IMRT* intensity-modulated photon-radiation therapy, *$* US dollars, *QALY* quality-adjusted life-year

^a^NTCP-reduction referred to the advantage of IMPT over IMRT in reducing symptomatic dysphagia and xerostomia, and calculated with the equation: NTCP-reduction (%) _=_ [(NTCP _after IMRT_ - NTCP _after IMPT_) / NTCP _after IMRT_] *100%.

^b^The willingness-to-pay threshold of China.
