# Supplementary material for: Optimizing oropharyngeal cancer management by using proton beam therapy: trends of cost-effectiveness
Source: BMC Cancer. 2021 Aug 21;21:944. doi: 10.1186/s12885-021-08638-2 (PMC8380358; doi:10.1186/s12885-021-08638-2)
Supplement: Supplementary file 5 — Additional file 5: Table S2 One-way sensitivity analysis identifying the cost-effective threshold value for NTCP-reduction. [file 12885_2021_8638_MOESM5_ESM.docx]

**Additional File 5: Table S2** One-way sensitivity analysis identifying the cost-effective threshold value for NTCP-reduction

| Age Level | Minimum NTCP-reduction for “Cost-effective”^a^ | | | | | | | | |
| --- | --- | --- | --- | --- | --- | --- | --- | --- | --- |
|  | Proton Treatment Cost of $50,000 | | | Proton Treatment Cost of $40,000 | | | Proton Treatment Cost of $30,000 | | |
|  | $33,558/QALY | $50,000/QALY | $100,000/QALY | $33,558/QALY | $50,000/QALY | $100,000/QALY | $33,558/QALY | $50,000/QALY | $100,000/QALY |
| 10-year-old | 47.5% | 35.8% | 20.6% | 35.3% | 26.6% | 15.2% | 22.9% | 17.2% | 9.8% |
| 20-year-old | 50.8% | 38.3% | 22.0% | 37.7% | 28.4% | 16.3% | 24.5% | 18.4% | 10.5% |
| 30-year-old | 55.6% | 41.9% | 24.1% | 41.3% | 31.1% | 17.8% | 26.8% | 20.2% | 11.5% |
| 40-year-old | 63.3% | 47.8% | 27.5% | 47.1% | 35.5% | 20.3% | 30.6% | 23.0% | 13.2% |
| 50-year-old | 77.2% | 58.3% | 33.5% | 57.6% | 43.4% | 24.9% | 37.5% | 28.2% | 16.1% |
| 60-year-old | -^c^ | 79.9% | 46.1% | 79.2% | 59.7% | 34.3% | 51.8% | 38.9% | 22.3% |
| 70-year-old | - | - | - | - | - | 66.8% | - | 76.6% | 43.8% |

*NTCP* normal tissue complication probability, *IMPT* intensity-modulated proton radiation therapy, *IMRT* intensity-modulated photon-radiation therapy, *$* US dollars, *WTP* willingness-to-pay, *QALY* quality-adjusted life-year

^a^NTCP-reduction referred to the advantage of IMPT over IMRT in reducing symptomatic dysphagia and xerostomia, and calculated with the equation: NTCP-reduction (%) _=_ [(NTCP _after IMRT_ - NTCP _after IMPT_) / NTCP _after IMRT_] *100%; IMPT becomes cost-effective when the NTCP-reduction is ≥ the corresponding value listed in the table.

^b^The willingness-to-pay threshold of China.

^c^The cost-effective scenario does not exist.
